# Image artifacts from MR-based attenuation correction in dedicated PET/MR breast coil for PET/MR mammography

**DOI:** 10.1186/2197-7364-2-S1-A62

**Published:** 2015-05-18

**Authors:** Ihnho Cho, Eunjung Kong, Kyunga Chun

**Affiliations:** Department of nuclear medicine, Yeungnam University Hospital, South Korea

We evaluated the artifacts in segmentation-based attenuation correction maps (μ-maps) of hybrid positron emission tomography/magnetic resonance (PET/MR) mammography using dedicated PET/MR breast coil in breast cancer patients.

## Materials and Methods

Attenuation map of hybrid F-18 FDG PET/MR mammography in 38 patients diagnosed with invasive breast carcinoma were retrospectively inspected for artifacts. The artifacts were subdivided into 2 groups with minor (group A) and major artifacts (group B) on the basis of their severity. The impact of μ-map artifacts on PET interpretation was evaluated qualitatively via visual analysis as well as quantitatively by comparing SUVmax of breast cancer between PET/MR mammography and whole body PET/MR.

## Results

Minor Attenuation map artifacts were found in 22 patients and major artifacts in 16 patients. Minor artifacts were field of view edge artifacts, lung boarder artifacts, small body contour artifacts, respiratory artifacts and trachea artifacts. Major artifacts were body contour artifact with missing dorsal body contour including both lungs (n=10), left lung (n=5) and wide expanded areas around breast and chest (n=1). All FDG-avid malignant mass were not affected by artifacts on visual PET interpretation. SUVmax in PET/MR mammography and whole body PET/MR in group A and B were 8.31±6.31, 6.15±4.20, 4.75±3.70 and 4.70±4.10, respectively. The changes in group A and B was 31.11% and 14.08%, respectively. Quantitatively, major μ-map artifacts led to significant SUVmax changes (p<0.001). No change in diagnosis was caused by μ-map artifacts. Major attenuation map artifacts that occur in a considerable percentage of hybrid PET/MR mammography have the potential to falsify PET quantification. However, there was no change in clinical diagnosis due to μ-map artifacts.

